# Supplementary material for: Baseline Characterization of the Gut Microbiota of Field and Colony Populations of Phlebotomus tobbi and Preliminary Assessment of the Anti-Leishmanial Activity of Cultivable Bacteria
Source: Pathogens. 2026 Jun 23;15(7):658. doi: 10.3390/pathogens15070658 (PMC13416292; doi:10.3390/pathogens15070658)
Supplement: Supplementary file 1 [file pathogens-15-00658-s001.zip › pathogens-4357168-File S1/Taxa2ASV_Decomposer/Family/f__Sphingomonadaceae/seqs.align.html]

```
seq58           AGTGGGGAATATTGGACAATGGGCGAAAGCCTGATCCAGCAATGCCGCGTGtGTGAaGAA
seq11           AGTGGGGAATATTGGACAATGGGCGAAAGCCTGATCCAGCAATGCCGCGTGAGTGATGAA
seq13           AGTGGGGAATATTGGACAATGGGCGcAAGCCTGATCCAGCAATGCCGCGTGAGTGATGAA
seq23           AGTGGGGAATATTGGACAATGGGCGAAAGCCTGATCCAGCAATGCCGCGTGAGTGATGAA
seq47           AGTGGGGAATATTGGACAATGGGCGAAAGCCTGATCCAGCAATGCCGCGTGAGTGATGAA
seq71           AGTGGGGAATATTGGACAATGGGCGAAAGCCTGATCCAGCAATGCCGCGTGAGTGATGAA
seq9            AGTGGGGAATATTGGACAATGGGCGAAAGCCTGATCCAGCAATGCCGCGTGAGTGATGAA
seq31           AGTGGGGAATATTGGACAATGGGCGAAAGCCTGATCCAGCAATGCCGCGTGAGTGATGAA

seq58           GGCCTTcGGGTTGTAAAGCaCTTTTgtCCGGaAaGAaAtggctctggttaatacctgggg
seq11           GGCCcTAGGGTTGTAAAGCTCTTTTACCCGGGAaGATA----------------------
seq13           GGCCcTAGGGTTGTAAAGCTCTTTTACCCGGGAaGATA----------------------
seq23           GGCCcTAGGGTTGTAAAGCTCTTTTACCCGGGATGATA----------------------
seq47           GGCCTTAGGGTTGTAAAGCTCTTTTACCCGGGATGATA----------------------
seq71           GGCCTTAGGGTTGTAAAGCTCTTTTACCCGGGATGATA----------------------
seq9            GGCCTTAGGGTTGTAAAGCTCTTTTACCCGGGATGATA----------------------
seq31           GGCCTTAGGGTTGTAAAGCTCTTTTACCCGGGATGATA----------------------

seq58           tcgATGACgGTACCGGaAGAATAAGgaCCGGCTAACTaCGTGCCAGCAGCCGCGGTAATA
seq11           ---ATGACtGTACCGGGAGAATAAGCcCCGGCTAACTCCGTGCCAGCAGCCGCGGTAATA
seq13           ---ATGACtGTACCGGGAGAATAAGCcCCGGCTAACTCCGTGCCAGCAGCCGCGGTAATA
seq23           ---ATGACAGTACCGGGAGAATAAGCcCCGGCTAACTCCGTGCCAGCAGCCGCGGTAATA
seq47           ---ATGACAGTACCGGGAGAATAAGCTCCGGCTAACTCCGTGCCAGCAGCCGCGGTAATA
seq71           ---ATGACAGTACCGGGAGAATAAGCTCCGGCTAACTCCGTGCCAGCAGCCGCGGTAATA
seq9            ---ATGACAGTACCGGGAGAATAAGCTCCGGCTAACTCCGTGCCAGCAGCCGCGGTAATA
seq31           ---ATGACAGTACCGGGAGAATAAGCTCCGGCTAACTCCGTGCCAGCAGCCGCGGTAATA

seq58           CGGAGGGgGCTAGCGTTGTTCGGAATTACTGGGCGTAAAGCGCACGTAGGCGGCTTTGTA
seq11           CGGAGGGgGCTAGCGTTGTTCGGAATTACTGGGCGTAAAGCGCACGTAGGCGGCTTTGTA
seq13           CGGAGGGgGCTAGCGTTGTTCGGAATTACTGGGCGTAAAGCGCACGTAGGCGGCTTTGTA
seq23           CGGAGGGgGCTAGCGTTGTTCGGAATTACTGGGCGTAAAGCGCACGTAGGCGGCTTTGTA
seq47           CGGAGGGAGCTAGCGTTGTTCGGAATTACTGGGCGTAAAGCGCACGTAGGCGGCTTTGTA
seq71           CGGAGGGAGCTAGCGTTGTTCGGAATTACTGGGCGTAAAGCGCACGTAGGCGGCTTTGTA
seq9            CGGAGGGAGCTAGCGTTaTTCGGAATTACTGGGCGTAAAGCGCACGTAGGCGGCTTTGTA
seq31           CGGAGGGAGCTAGCGTTGTTCGGAATTACTGGGCGTAAAGCGCACGTAGGCGGCTTTGTA

seq58           AGTCAGAGGTGAAAGCCTGGAGCTCAACTCCAGAACTGCCTTTgAGACTGCATCGCTTGA
seq11           AGTCAGAGGTGAAAGCCTGGAGCTCAACTCCAGAACTGCCTTTgAGACTGCATCGCTTGA
seq13           AGTCAGAGGTGAAAGCCTGGAGCTCAACTCCAGAACTGCCTTTgAGACTGCATCGCTTGA
seq23           AGTtAGAGGTGAAAGCCTGGAGCTtAACTCCAGAACTGCCTTTAAGACTGCATCGCTTGA
seq47           AGTCAGgGGTGAAAGCCTGGAGCTCAACTCCAGAACTGCCTTTgAGACTGCATCGCTTGA
seq71           AGTCAGgGGTGAAAGCCTGGAGCTCAACTCCAGAACTGCCTTTAAGACTGCATCGCTTGA
seq9            AGTtAGAGGTGAAAGCCTGGAGCTCAACTCCAGAAtTGCCTTTAAGACTGCATCGCTTGA
seq31           AGTtAGAGGTGAAAGCCTGGAGCTCAACTCCAGAACTGCCTTTAAGACTGCATCGCTTGA

seq58           ATCCAGGAGAGGTcAGTGGAATTCCGAGTGTAGAGGTGAAATTCGTAGATATTCGGAAGA
seq11           ATCCAGGAGAGGTcAGTGGAATTCCGAGTGTAGAGGTGAAATTCGTAGATATTCGGAAGA
seq13           ATCCAGGAGAGGTcAGTGGAATTCCGAGTGTAGAGGTGAAATTCGTAGATATTCGGAAGA
seq23           ATCCgGGAGAGGTGAGTGGAATTCCGAGTGTAGAGGTGAAATTCGTAGATATTCGGAAGA
seq47           ATCCgGGAGAGGTGAGTGGAATTCCGAGTGTAGAGGTGAAATTCGTAGATATTCGGAAGA
seq71           ATCCgGGAGAGGTGAGTGGAATTCCGAGTGTAGAGGTGAAATTCGTAGATATTCGGAAGA
seq9            ATCCAGGAGAGGTGAGTGGAATTCCGAGTGTAGAGGTGAAATTCGTAGATATTCGGAAGA
seq31           ATCCAGGAGAGGTGAGTGGAATTCCGAGTGTAGAGGTGAAATTCGTAGATATTCGGAAGA

seq58           ACACCAGTGGCGAAGGCGGCTgACTGGACTGGTATTGACGCTGAGGTGCGAAAGCGTGGG
seq11           ACACCAGTGGCGAAGGCGGCTgACTGGACTGGTATTGACGCTGAGGTGCGAAAGCGTGGG
seq13           ACACCAGTGGCGAAGGCGGCTgACTGGACTGGTATTGACGCTGAGGTGCGAAAGCGTGGG
seq23           ACACCAGTGGCGAAGGCGGCTCACTGGACcGGTATTGACGCTGAGGTGCGAAAGCGTGGG
seq47           ACACCAGTGGCGAAGGCGGCTCACTGGACcGGTATTGACGCTGAGGTGCGAAAGCGTGGG
seq71           ACACCAGTGGCGAAGGCGGCTCACTGGACcGGTATTGACGCTGAGGTGCGAAAGCGTGGG
seq9            ACACCAGTGGCGAAGGCGGCTCACTGGACTGGTATTGACGCTGAGGTGCGAAAGCGTGGG
seq31           ACACCAGTGGCGAAGGCGGCTCACTGGACTGGTATTGACGCTGAGGTGCGAAAGCGTGGG

seq58           GAGCAAA
seq11           GAGCAAA
seq13           GAGCAAA
seq23           GAGCAAA
seq47           GAGCAAA
seq71           GAGCAAA
seq9            GAGCAAA
seq31           GAGCAAA
```
